# Tendency to Vacuum in the Cerebro-Spinal Cavity

**Published:** 1861-01

**Authors:** Daniel Brainard

**Affiliations:** Professor of Surgery in Rush Medical College


### SUGGESTIONS ON THE TENDENCY TO VACUUM

### IN THE CEREBRO-SPINAL CAVITY WHICH EXISTS IN CERTAIN SPASMODIC DISEASES.

BX DANIEL BRAINARD, M. D.,

*Professor of Surgery in Rush Medical College.*

I. Since the discovery of the "cerebro-spinal" fluid, by Magendie, and the description of the veins of the head and spinal column by Breschet, the opinion has been gaining ground, that these vessels and this fluid have for their object in part the regulation of the degree of pressure exerted upon the nervous centres and obviating the effects of sudden and even of gradual changes.

Active as the circulation of the brain and spinal cord are known to be, it is impossible to attribute the extensive nervous plexuses which exist around and within the cranium and spinal column, to the wants of the circulation alone.

It is not my intention to describe these veins, which has been done so completely by the recent anatomists whose works are in the hands of students, particularly by Cruveillhier, Quain and Wilson; but there are some points to which I desire to call attention, as bearing especially on the objects of this essay.

Of these, the first is that the cerebral and spinal veins constitute a separate system which, in its independence, may be compared to the portal system. Thus, the veins of the spine, from the extremity of the sacrum to the top of the dorsal division, (but most perfectly in this latter,) are collected into the venæ azygos, which, instead of passing into the ascending cava, empties into the descending cava, through the medium of the venæ innominatæ and the subclavian vein, where are gathered, all the principal venous trunks emanating from thes interior of the cerebro-spinal cavity.

In the second place, it is important to remember that the venæ azygos are without valves,* and that the circulation

* Cruveillhier, Descriptive Anatomy.

through them is so free, that the ligation of all the jugular veins does not prevent the circulation of the brain from being carried on, as Cruveillhier has proved by experiment.*

It should also be borne in mind in all studies on the circulation, that the coats of the spinal venous plexuses are not only very thin, but that "the muscular element is wanting in most of the veins of the brain and pia mater."

These facts are cited in order to facilitate the apprehension of the manner and degree in which the respiratory movements act upon the brain, and influence its movements, its states of fullness and of vacuity.

II. Representing the prevailing opinions on the subject of pressure on the brain, I quote from the following works of authority:

"Magendie's experiments show that the brain and spinal cord are surrounded by fluid, the pressure of which probably antagonizes that which must be exerted through the blood vessels." "This fluid is a valuable regulator of vascular fullness within the cranium, and a protection of the brain against too much pressure from within." †

"The amount of this cerebro-spinal fluid seems to average about two ounces; but in case of atrophy of the brain, as much as twelve ounces of fluid may sometimes be obtained from the cranio-spinal cavity." "It evidently serves as an equalizer of the amount of pressure within the cranial cavity, admitting the distension or contraction of the vessels to take place within certain limits without any considerable change in the compression to which the nervous matter is subjected." ‡

It may be added, that while the above quotations express the prevailing opinions of physiologists, the experiments of

* Kolliker, Manual of Microscop. Anat. p. 689.

† Todd and Bowman, Physiological Anatomy and Physiology of Man, Am. Ed. p. 268.

‡ Carpenter, Human Physiology, Am. Ed. p. 580.

Longet and Cruveillhier seem to show that the importance of this fluid to life has been over-estimated by Magendie.‡

The discussion in regard to the brain being subject to atmospheric pressure appears to be settled in the affirmative. As the veins situated on the exterior of the cavity communicate freely with those of the interior, it follows that through their medium the pressure of the atmosphere must be exerted to some degree on the brain, unless it be obviated in some manner. The only way in which this can be accomplished, according to our present knowledge, is by the act of inspiration, in which the vacuum produced in the thorax extends in the same degree to the cavity of the spine and cranium, causing the sinking of the cerebral surface, which is so familiar to physiologists.

The heart at the same time propels the arterial blood with a certain force into the cranial cavity. The amount of this force has been very accurately measured by the instrument of Poisseuille, and has been found to vary at different times according to the degree of fullness of the vessels, during inspiration and expiration, etc. This force of the heart's action, super-added to that of the atmosphere on the veins without the spine, would seem sufficient to cause a certain degree of pressure within the cranium, and such is the veiw generally received. But although the power of the heart's action has been very accurately measured, the resisting power of the arteries and capillaries has not, and as the arteries are not only elastic, but muscular, and contract with various degrees of force at various times, it is impossible correctly to estimate the degree in which the impulse of the blood is met and resisted by the bony walls of the skull. Nor does the experiment of opening the cranium afford much light on this point, as the operation itself causes in most cases an alteration of the respiration, which increases the pressure on the brain.

‡ For a pretty full discussion on this subject, refer to Burrows, on the Disorders of the Cerebral Circulation.

Burrows, who labors to show that this pressure is constant and considerable, seems to me not to have succeeded so perfectly as might be desired in regard to so important a point, and I think that the results of the experiments of trephining animals and observations of cases of exfoliations of considerable portions of the cranium tend to show that in a state of health, repose, and *waking*, the pressure from within very nearly balances that from without.

III. Whatever difference of opinion may exist in regard to that subject, there can be none in reference to the fact that in many abnormal states, the pressure on the brain from within does greatly predominate in such a degree as to give rise to various symptoms of disease, and even to cause death.

I propose here to notice briefly the effects of pressure on the brain and cord.

These are different, according to the degree, the extent and the suddenness of the pressure.

Marshall Hall* considered the suggestion rather as a conjecture than a well matured opinion, that sleep is caused by a slight degree of physiological pressure on the brain. He suggests, that by some muscular arrangement, the return of venous blood is interrupted in a very slight degree, and the proper degree of pressure is thus produced. This view of Marshall Hall has not received the attention which it seems to me to merit. Every one is familiar with the appearances the swelling of the veins and tissues of the face, the stertor which indicate increase of pressure on the brain during sleep and obstruction of the circulation. Long before being aware of Marshall Hall's opinion, I had arrived at the same conclusion from different premises.

I observed that in subjects affected with spina bifida and hydrocephalus, the tumor in the one case and the head in the other, were tense during sleep and lax in waking. I noticed also, that after tapping, the subjects either slept little and with

* "Pressure on the brain produces at once functional inactivity."

agitated sleep, or not at all, so long as the sac remained very lax, but soundly and even with stertor when the pressure was increased to a certain point.

Pressure on the brain, whether applied by accidental effusion or otherwise, if carried to a certain point, produces loss of function; on the cerebrum, it produces insensibility and loss of voluntary movements.*

This effect is not disputed, and not only physiological, but surgical works abound with facts in proof.† The exact manner in which pressure acts, is, however, demonstrated very clearly by Flourens, to whose work‡ I refer for more ample information. The work of Burrows already cited contains much valuable information on the subject of pressure on the brain. He shows that a very considerable effusion may exist in the cranial cavity without producing insensibility.

The effects of pressure in producing congestions, sanguineous and serous effusions, have been studied by Andral, Abercrombie, Rokitansky and other pathological anatomists in so perfect a manner as to embrace nearly every variety of pathological changes likely to occur. The true interpretation of many of these appearances remains to be given, and all the symptoms of compression, and even death, occur in some cases without any mechanical cause being discovered after death, sufficient to explain them.

IV. *Want of Pressure on the Brain.*—Want of pressure on the brain, from loss of blood, from anæmia, from sudden cessation of the heart's action, etc., produces that suspension of the function of the brain called syncope. This is, I think, undoubtedly the true cause, although there are not wanting those who regard it as due to congestion. It is incredible that

* "If an aperture be made in the skull, and the protruding portion of the brain be subjected to pressure, the immediate suspension of the activity of the whole organ is the result."

† Carpenter, Physiology, p. 530, and Draper, Human Physiology, p. 326.

‡ Experimental researches on the properties and functions of the nervous system, Paris, 1842.

insensibility, on assuming the erect posture by a person who has suffered from hemorrhage, can be produced by the fullness of the cerebral vessels.

In syncope, consciousness and voluntary movements are lost, and the heart's action is feeble or suspended. In this latter symptom, it differs from coma and apoplexy, in which the action of the heart continues with force, although more slowly than natural. If the syncope be prolonged or perfect, spasms usually occur and death from loss of blood is always attended with convulsions. This fact has been particularly insisted upon by Marshall Hall and C. Bernard.

Rokitansky has pointed out very clearly the effects as well as the causes of vacuum in some states. (Path. Anat., vol. 3, p. 287 *et seq.*) "The vacuum within the skull, produced by shrinking of the brain, is filled up principally by a clear colorless serum which accumulates in the sac of the arachnoid around the brain, in the tissue of the pia mater and in the ventricles." This form of atrophy he states to be very frequent in old age, delirium tremens and mental diseases attended by excitement. "It is in itself a very important condition, but it becomes still more so from its immediate and further consequences. These consequences are as follows:

1st. Congestion of the brain, *Hyperæmia ex vacuo.* These congestions give rise to transient or protracted attacks, which simulate apoplexy and are so frequent in old age.

2nd. Actual apoplexy.

3rd. Œdema of the brain.

"Atrophy of an entire hemisphere of the cerebrum, produced in this manner, is sometimes concentric; sometimes its lateral ventricle is dilated, then it is excentric."

The possible existence of so strong a tendency to vacuum in spasmodic diseases as to give rise to rupture of vessels and effusion, seems not to have been suspected by Rokitansky.

Marshall Hall was the first in England to show that what was called Hydrocephalus, but which he called Hydrocephaloid disease, depended not upon fullness, but upon deficient circulation. In his work "on the Diseases and Derangements of the Venous System," p. 154, he says, "This question and that of the effects of exhaustion in infants and children, open a new field of investigation." "The subject must be taken up and investigated anew." "In this manner, some hydrocephaloid, convulsive, and even croupy affections, will be received in a new aspect."

The tendency to *vacuum*, as a cause or an accompaniment of convulsive diseases, has never, as far as I am aware, been suspected, notwithstanding that facts pointing directly to it have not unfrequently been observed. The first disease in which it has been shown to exist (without being suspected) is tetanus of new born infants.

Dr. Marion Sims first pointed out (Am. Journal, April, 1854, p. 363) that in many cases the cranial bones are overlapped, the occipital being depressed. He attributed this to the position of the child and pressure, a theory which further observations have hardly confirmed.

In the same periodical of October, 1858, p. 354, Dr. Sims, after further observations, states that in some cases the parietal bones were depressed, while in others the depression of the occipital was so slight as to require a particular method of examination to detect it.

Dr. Wm. A. Baldwin (Am. Journal for Oct., 1846,) states that in some cases of this disease he found no depression of the bones. Drs. Wooter and F. W. Clement have found this depression in cases observed by them.

This displacement has been so often noticed, that its frequent occurrence cannot be doubted. It is not however always present, nor is its presence necessarily attended with trismus. Since the first publication of Dr. Sims' views, I have often observed this overlapping in new born infants. It generally disappeared when the respiration became well established. In one case in which hemorrhage occurred, it lasted for several weeks. I have seen but two cases of tetanus in infants; in one of these there was overlapping; in the other, the child being older, the sutures were formed so that the bones could not overlap, but in this case the anterior fontanelle was depressed particularly during the paroxysm of spasm. This appearance was sometimes noticed in convulsions in children, but I have not as yet been able to determine the circumstances in which it is present.

I conclude from these facts that the overlapping of the cranial bones in infants is, excepting that which is temporarily produced by pressure during labor, the result of want of support within, and that in this form of spasm the cerebro-spinal cavity is exhausted so that these bones are all forced inward by the pressure of the atmosphere.

That this is the true theory of this displacement, seems to be pretty clearly shown by a statement of Dr. Sims, that he, in one instance, raised up the occiput with a sharp instrument after death, and that, unless supported by pressure on the side of the head, it returned to its position with force.

The mechanical means by which this want of support is brought about, must be referred to feeble action of the heart or a peculiar modification of the respiratory movements, or both. If a strong effort at inspiration be made while the opening of the larynx is closed perfectly or partially, the effect will be to exhaust the cavity of the cranium and that of the spine. The croupy and sibilant sound often heard from the respiration of tetanic patients, shows that this closure in a degree does often exist.

The appearances, on dissection, are best explained by this theory. The most constant of these, is an effusion of blood into the sheath of the spinal cord. This may exist with a thick gelatinous effusion, a swelling of the roots and ganglia of the spinal nerves, without any signs of an inflamatory affection. In some cases, no appearances have been found in the nervous centres to account for death.

There is strong reason to believe that trismus of new born infants does not differ in cause or character, in general, from traumatic tetanus. Now, one of the most constant and distressing symptoms of tetanus is known to be wakefulness; there is an impossibility of procuring sleep. Mr. Curling remarks, during the height of the disease, *it appears impossible for sleep to occur*, and if it does, the muscles are relaxed. He remarks, (p. 152 Treatise on Tetanus,) "In tetanus, as well as in hydrophobia and delirium tremens, there appears to be a conventional license for the exhibition of opium. A drachm or two drachms of laudanum, or a scruple of solid opium every three or four hours, are ordinary doses."

"The pupil is generally found contracted in tetanus."

In this disease, in hydrophobia, in certain forms of insanity, in spasmodic cholera and delirium tremens, whenever prolonged vigilance exists, there must be not only a want of normal pressure on the brain, but a peculiar action is exerted by which the physiological pressure which causes sleep and that which gives rise to the coma from opium, are absolutely prevented.

Epilepsy. — The turgescence of the vessels of the face during epileptic paroxysms and the somnolence which follows them, seem to indicate beyond all doubt, that fullness of the vessels and increased pressure on the brain, generally exist in certain stages; yet it has often been noticed that great pallor precedes the paroxysm, and Dr. Brown Sequard has, I think, attributed the attack to spasm of the arteries of the brain. I have had an opportunity which has not perhaps been offered to any other surgeon, of observing the condition of the brain in the human subject, during an epileptic paroxysm.

On the 18th January, 1860, I trephined a patient in the Chicago City Hospital for an old depression of the skull. The patient was 29 years old; sixteen years previously he received the kick of a horse on the right side of the head, about one inch above the ear, causing a deep depression of the bone. It was noticed that soon after his character changed and he became morose and taciturn. Three years after this accident, he had the first severe epileptic paroxysm. They gradually increased in frequency until the time when he came under my care, when they were so frequent during the day as to be numbered with difficulty.

The patient was in a state of dementia and incapable of answering qustions intelligibly. He was put under chloroform, and the depressed portion of bone removed by two applications of the crown of the trephine. A sharp edge of bone had projected through the dura mater, so that an opening in this membrane was caused by its removal. At first, the surface of the cerebrum was pressed gently against the opening, but while I was waiting for a slight bleeding to stop, and wiping away the blood with a sponge, the patient was seized with an epileptic spasm. He made a long, deep inspiration, with a hissing sound. At the instant, there was a gush of serum from the wound, and on putting my finger into it to ascertain the source of this discharge, I found the surface of the cerebrum so much depressed, that the finger could be passed between it and the dura mater with ease. The spasm continued for about two minutes, during which time the surface of the brain rose during expiration, but not so as to fill the cavity, and sank during the inspirations, as at first.

For twenty-four hours, the patient seemed doing well, answered some questions and called one of his friends by name, which he had not done before for a long time; but at the end of that time, he took a spasm which lasted an hour and left him somnolent. He died fifty-eight hours after the operation.

The head was examined seven hours after death. On raising the piece of scalp which had been circumscribed by the incisions, and passing the finger into the wound, it passed di rectly into the lateral ventricle of the right side. On removing the vault of the cranium the pia mater was found injected with slight opalescent appearance of the arachnoid. The substance of the brain was firm. The left lateral ventricle contained two fluid ounces of serum, the right was filled with imperfectly formed pus. The examination was not allowed to go further. It however revealed the cause of the escape of the serum from the wound, during the spasm after the operation. It came from the lateral ventricle, which was ruptured by the pressure of the atmosphere during an inspiration with the larynx nearly closed. How far the dilatation of the ventricles may be attributed to a tendency to vacuum during the paroxysms or to atrophy of the brain taking place gradually as described by Rokitansky, I will not presume to say. The case affords a striking proof of the extent to which a vacuum may be produced in the cranium by spasmodic respiration.

Nor need I stop to explain the mechanism by which such movements are capable of producing appearances of congestion in the vessels, effusions of serum, hemorrhage from rupture of the vessels, etc. I will only add, that in dogs killed with strychnine after trephining, there has seemed to be a considerable pressure on the brain, and no tendency to vacuum.

As the object of this notice is simply to draw the attention of those who have fields of observation to the subject, the treatment which these views might suggest will be deferred to a subsequent paper. It may be seen readily that the plan of Marshall Hall of performing tracheotomy in tetanus hydrophobia, etc., has relation to the points in question, and that the futility of the usual treatment of tetanus taken in connexion with the views herein advanced, naturally points to stimulation, as the treatment most likely to be successful.

Experience has already led several eminent surgeons to adopt this course, among whom may be named Dr. Carmichael. He some years since published, in the Dublin Hospital Reports, some cases treated by the ordinary method with the usual results, and added, that in view of its generally fatal character he should not hesitate thereafter to resort to the use of stimulants.

Note.—In view of the almost universal fatality of trismus nascentiun and tetanus in warm climates, and the comparatively rare occurrence of these cases in this latitude, which prevents a trial of any plan on an extensive scale, I take the liberty of asking the attention of those whose situation for observation is more favorable than my own to the following suggestions concerning the method of production and the treatment, especially of trismus of infants.

It is possible that the disease is produced by an animal poison similar to but differing from that of hydrophobia. This poison may be generated in the wound, absorbed into the blood vessels, and at varying periods, and in different degrees, produce the disease.

Considering the neutralizing properties of iodine and chlorine for all putrescent and some other poisons, it is likely that one or the other of these substances, but especially the iodine, it being the less active, may be the best preventive and even curative application. Used in substance upon the surface of the body (protected from its action) and covered with oiled silk or other impervious tissue, it rises in vapor, is speedily absorbed, as its presence in the urine shows. Its application over the umbilicus might be the means of preventing trismus, although I should hardly venture to hope it would cure it when once developed to a severe degree.

